# Effects of Different Living Environments on Intestinal Flora in Patients With *Schistosoma Japonicum‐Induced Liver Fibrosis*

**DOI:** 10.1002/mbo3.70366

**Published:** 2026-08-04

**Authors:** Xiaofei Fan, Chen Zhou, Pengpeng Zhang, Yingzi Ming

**Affiliations:** ^1^ Shandong Medical College Jinan China; ^2^ Cardio‐thoracic Surgery Center, Changsha Central Hospital University of South China Changsha China; ^3^ Transplantation Center, Engineering and Technology Research Center for Transplantation Medicine of National Health Comission, The Third Xiangya Hospital Central South University Changsha China

**Keywords:** cirrhosis, gut microbiome, infected water, *Schistosoma japonicum* infection

## Abstract

*Schistosomiasis japonica* is a parasitic disease leading to liver cirrhosis. China's “fishermen going ashore” policy divides patients with schistosomiasis liver fibrosis into two groups: those near the infected waters and those inland far from the infected water. This study aims to compare the differences in intestinal flora between two groups from the perspective of intestinal flora, and provide a basis for future prevention and control priorities. This study collected feces and basic information of patients with *Schistosoma japonicum* cirrhosis living near infected water and living on land. The characteristics of intestinal flora of the two types of patients were compared by 16sRNA sequencing technology. The infected water contact group and the terrestrial living group showed significant differences in intestinal flora characteristics: the former showed dominance of *Firmicutes*, high α‐diversity, enrichment of butyrate‐producing bacteria (such as *Blautia*), and enhanced environmental adaptability; the latter showed an imbalanced state with increased Proteobacteria and reduced α‐diversity, accompanied by abnormal lipid metabolism and barrier function damage.

## Graphical abstract

Different living environments have a significant impact on the gut microbiota of patients with *Schistosoma japonicum*.

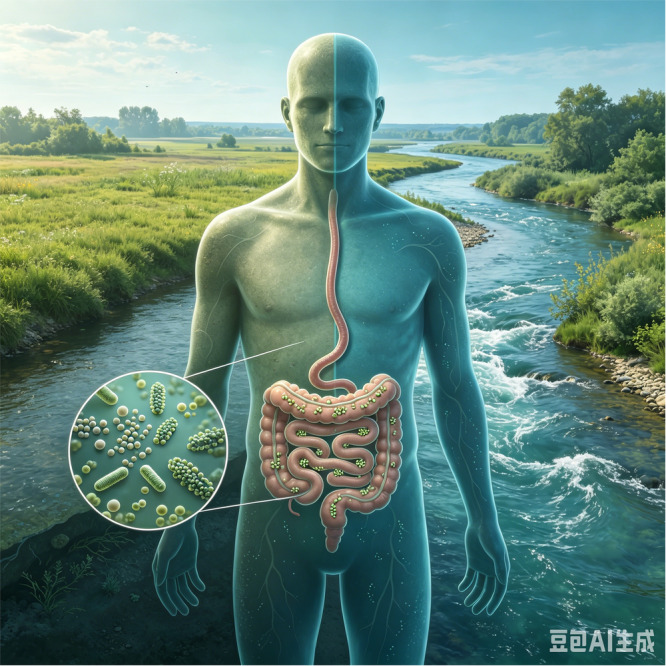

## 1 Background

*Schistosomiasis japonica* is an important zoonotic parasitic disease and one of the neglected tropical diseases (NTDs) defined by the World Health Organization (WHO) (Zumuk et al. 2024). At present, the disease is mainly prevalent in East Asia and Southeast Asia, including China, the Philippines and Sulawesi Island in Indonesia. Among them, the Yangtze River Basin in China and the lakes and swamps to its south were once historically high‐incidence areas (Jain 2025). According to WHO statistics in 2024, at least 253.7 million people required preventive treatment for schistosomiasis in 2024, out of which more than 100.5 million people were reported to have been treated (Liang et al. 2011).

Chronic schistosomiasis infection often leads to periportal liver fibrosis, and this pathological process may have a two‐way regulatory relationship with intestinal flora disorders (Lu et al. 2024). Schistosomiasis infection can promote flora translocation by destroying the intestinal mucosal barrier (Zhou et al. 2023). The metabolites of specific intestinal microorganisms may affect the process of liver fibrosis by regulating the host immune response (Zhou et al. 2022). This mechanism provides an important perspective for exploring new intervention strategies for schistosomiasis cirrhosis.

China was once one of the countries with the most serious epidemics of *Schistosomiasis japonica* (Li et al. 2014). In the comprehensive strategy of schistosomiasis prevention and control in China, “fishermen going ashore” (or “fishermen changing their jobs”) is a key environmental intervention and social security measure, mainly targeting fishermen who have been living on boats for a long time and frequently in contact with infected water (Ross et al. 1997). This group is exposed to the risk of schistosomiasis infection all year round due to the characteristics of their occupation. With the promulgation of the “Regulations on the Prevention and Control of Schistosomiasis” (1990), a large number of fishermen began to migrate from infected waters to inland areas far away from infected waters (Huang and Manderson 2005). Since then, Chinese patients with *Schistosoma japonicum*‐induced liver fibrosis have gradually differentiated into those living in an environment close to infected waters and those living inland far away from infected waters. Individuals residing along rivers or lakeshores exhibit persistent, high‐frequency, and often perennial exposure to cercariae‐infested water due to prolonged contact with contaminated water bodies. In contrast, in inland endemic areas, water bodies are relatively scattered and limited in scale, and the distribution of snail intermediate hosts is uneven. Consequently, water contact among inland residents is intermittent and seasonal, with generally low exposure intensity. These geographical differences may lead to distinct infection patterns. Therefore, although both groups of patients suffered from the same diseasE—Schistosomiasis japonicus infection, as the disease progressed, the patient population living along the river showed a more severe clinical phenotype: more severe cirrhosis, more frequent abdominal pain and diarrhea, higher fecal oocyte detection rate and serum antigen positivity rate, and a significant increase in the number of previous praziquantel treatments.

The “fishermen going ashore” policy has played an important role in curbing the prevalence of schistosomiasis, effectively curbing the spread of schistosomiasis and reducing new infectious diseases (Leonardo et al. 2002). Currently, no research reports whether these two drastically different environmental exposure backgrounds shape differentiated gut microbiota characteristics, or whether such differences are related to different long‐term outcomes in patients. Therefore, our study, for the first time, used patients with schistosomiasis japonicus living “near the infected water” and “on land” as research subjects to systematically analyze the structural and functional differences in the gut microbiota between the two groups.

## 2 Methods

### 2.1 Study Design

This study strictly followed the inclusion and exclusion criteria to recruit patients with *Schistosoma japonicum*‐induced liver fibrosis. According to the living environment and region, the participants were divided into a living near the infected water group (WSJ) and living on land group (LSJ). The recruitment of patients of LSJ group was completed at the Third People's Hospital of Hunan Province, China from October 2021 to October 2022 (*n* = 8). Patients of WSJ group were recruited during an epidemiological survey in May 2022 (*n* = 10). The study followed the World Medical Association's Declaration of Helsinki and was approved by the Clinical Research Ethics Committee of the Third XiangYa Hospital of Central South University (Ethics Approval Number: Fast I 21145). All participants signed informed consent forms and their privacy was strictly protected. Inclusion and exclusion criteria were shown in Table 1. Basic information of groups was shown in Table 2.

**Table 1:** Inclusion and exclusion criteria.

Group	Inclusion criteria	Exclusion criteria
WSJ	1. Living in an epidemic area or having a history of contact with epidemic water for many times. 2. Schistosoma eggs were found in fecal examination or serological examination was positive. 3. B‐ultrasound showed hepatic fibrosis. 4. Living near infected water for the past 20 years. 5. 35–65 years old. 6. Positive serological test for schistosomiasis.	1. History of cancer. 2. History of chronic diseases such as hypertension and diabetes. 3. History of heart disease, hepatitis, or kidney disease. 4. Acute infection state. 5. Use of antibiotics within the past six months. 6. Use of proton pump inhibitors in the past six months.
LSJ	1. Living in an epidemic area or having a history of contact with epidemic water for many times. 2. Schistosoma eggs were found in fecal examination or serological examination was positive. 3. B‐ultrasound showed hepatic fibrosis. 4. Living on land far away from infected water for the past 20 years. 5. 35–65 years old. 6. Positive serological test for schistosomiasis.	1. History of cancer. 2. Acute infection state. 3. Patients infected with hepatitis A, B, C, D and E viruses. 4. The patient infected with other parasitic diseases or infectious diseases. 5. Use of antibiotics within the past six months. 6. Use of proton pump inhibitors in the past six months.

**Table 2:** Basic information of study population per group.

Variables	WSJ	LSJ	Difference
*n*	10	8	
Age (years)	55 ± 3.24	55 ± 3.28	0.7862
Gender (male/female)	3/7	4/4	0.2639
Active smoker (%)	40	12.5	0.1956
Active drinker (%)	30	25	0.816
Dietary type	Eastern diet	Eastern diet	No
	100%	100%	

*Note:* There were no statistically significant differences among three groups. Differences in age were determined using *t*‐test, *p* = 0.7862. Differences in gender, active smoker and drinker were determined using the Chi‐square test, χ2 of gender = 0.7481, df = 1 χ2 of smoker= 1.6751, df = 1, χ2 of drinker = 0.054, df = 1. *WSJ* Patients with Schistosoma japonicum liver fibrosis living near the infected water, *LSJ* Patients with Schistosoma japonicum liver fibrosis living on land.

Before sample collection, all subjects received professional training. Fresh fecal samples were placed in sterile collection tubes within half an hour after collection, quickly frozen in liquid nitrogen for 20 min, transferred to cryopreservation tubes, and stored in a −80°C refrigerator. After all samples of the same research group were collected, subsequent intestinal flora sequencing and data analysis were performed.

After a 12‐h fast, venous blood samples were collected from the participants in the early morning. White blood cell count (WBC), hemoglobin (Hb), and platelet count (PLT) were measured using an automated hematology analyzer (XE‐5000, Sysmex). Alanine aminotransferase (ALT), aspartate aminotransferase (AST), and albumin (ALB) were measured using an automated biochemical analyzer (Beckman Colter).

All subjects underwent abdominal color Doppler ultrasound examination. The degree of liver parenchymal fibrosis was assessed by an experienced sonographer according to the Niamey classification recommended by the World Health Organization (WHO) and the Special Program for Research in Tropical Diseases (TDR). Based on the thickening of the portal vein wall and the morphology and density of the echogenic reticular pattern of the liver parenchyma, the degree of liver fibrosis was classified into grade 0 (no reticular pattern, homogeneous echo), grade I (few punctate/short linear echoes, unevenly distributed), grade II (typical fish‐scale/reticular echoes, reticular pattern < 3 mm), and grade III (typical turtle‐back/map‐like echoes, reticular pattern > 3 mm, with or without portal vein dilation and splenomegaly).

### 2.2 Genomic DNA Extraction and 16S rRNA Gene Amplification

1. **DNA extraction:**
  The total genomic DNA of the samples was extracted using the CTAB method combined with a magnetic soil and feces DNA extraction kit (TIANGEN DP712). The DNA quality was tested by 1.0% agarose gel electrophoresis, and qualified samples were diluted to a working concentration of 1 ng/μl.
2. **Amplification of the V3‐V4 region of the 16S rRNA gene:**

PCR amplification was performed using specific primers 341 F (5′‐CCTACGGGNGGCWGCAG‐3′) and 806 R (5′‐GGACTACHVGGGTATCTAAT‐3′). The reaction system (30 μl) contains:

- Phusion High‐Fidelity PCR Master Mix 15 μl
- 0.2 μM each of forward and reverse primers
- Template DNA approximately 10 ng

Amplification procedure:

Preliminary denaturation at 98°C for 1 min;

30 cycles (98°C for 10 s, 50°C for 30 s, 72°C for 60 s);

Final extension at 72°C for 5 min.

### 2.3 PCR Product Detection and Sequencing Library Construction

The PCR products were mixed with SYBR Green‐stained 1× loading buffer and detected by 2.0% agarose gel electrophoresis to screen samples with clear main bands and a size range of 400–450 bp. The samples were purified using the AxyPrep DNA Gel Extraction Kit (AXYGEN). The sequencing library was then constructed using the NEB Next Ultra DNA Library Prep Kit for Illumina (NEB), and the index sequence was added according to the standard process. Finally, double‐end sequencing (read length 250/300 bp) was performed on the Illumina Miseq/HiSeq. 2500 platform.

### 2.4 Data Analysis

The sequencing data were analyzed using standard bioinformatics processes. First, the original double‐end sequencing reads were spliced using FLASH software (v1.2.11), and then OTU clustering was performed using the UPARSE pipeline (v7.0.1001) with a 97% similarity threshold, and species annotation was performed using the RDP classifier (v2.2) (confidence threshold 0.8). Diversity analysis included: using QIIME (v1.9.1) to calculate the alpha diversity index (Chao1, Shannon, etc.), ANOSIM analysis based on the Bray‐Curtis distance matrix to evaluate the differences between groups, and the LEfSe algorithm (LDA score > 2.0) to identify characteristic microbial markers between groups. All analyzes used default parameters, and data visualization was completed using the R language (v3.6.3).

## 3 Result

We first compared the disease phenotypes, including cirrhosis, between the two groups. Liver ultrasound revealed a higher grade of liver fibrosis in the WSJ group, with more patients exhibiting reticular changes in the liver. We then compared liver function test results. While there was no significant difference in ALT levels between the two groups, serum albumin levels were significantly lower in the WSJ group. Further comparison of complete blood counts showed no difference in white blood cell count or hemoglobin levels, but the WSJ group had lower platelet levels compared to the LSJ group. To assess the infection burden, we performed egg counts. The WSJ group showed a higher egg load in feces, but no eggs were found in the feces of the LSJ group in our study. Further epidemiological investigation revealed that patients in the WSJ group received more praziquantel treatments during disease progression. Additionally, patients in the WSJ group experienced more episodes of acute abdominal pain and diarrhea within 1 year (Table 3). Overall, our comparison suggests that patients living near flooded areas had higher levels of liver fibrosis, poorer liver function, coagulation function, and nutritional status.

**Table 3:** Information of phenotypic differences of per group.

Variables	WSJ (*n* = 10)	LSJ (*n* = 8)	*p*	Z/T
Fecalegg count	2 (0, 4)	0	0.0053	3.117
WBC (*10^9^/L)	6.05 (3.521, 8.564)	5.169 (4.321, 7.541)	0.5148	0.67
HB (g/L)	94 (87, 104)	96 (89, 110)	0.8817	0.54
PLT (*10^9^/L)	164 (111, 189)	188 (154, 224)	0.0458	2.19
ALT (U/L)	30 (17, 37)	25 (19, 31)	0.0794	0.42
AST (U/L)	32 (23, 42)	24 (19, 32)	0.0206	2.516
ALB (g/L)	30 (27, 38)	35 (28, 42)	0.0126	2.817
Liver fibrosis grade 0	0/10	0/10	—	—
Liver fibrosis grade I	2/10	4/8	—	—
Liver fibrosis grade II	5/10	3/8	—	—
Liver fibrosis grade III	3/10	1/8	—	—
Praziquantel treatment	2 (1, 3)	1 (1, 2)	0.043	2.02
Abdominal pain and diarrhea	3 (1, 7)	1.5 (1, 3)	0.0294	2.316
Liver reticular change	8/10	4/8	—	—

Abbreviations: ALB, albumin; ALT, alanine aminotransferase; AST, aspartate aminotransferase; HB, hemoglobin; PLT, platelets; WBC, white blood cell count.

3.1

**Figure 1:**
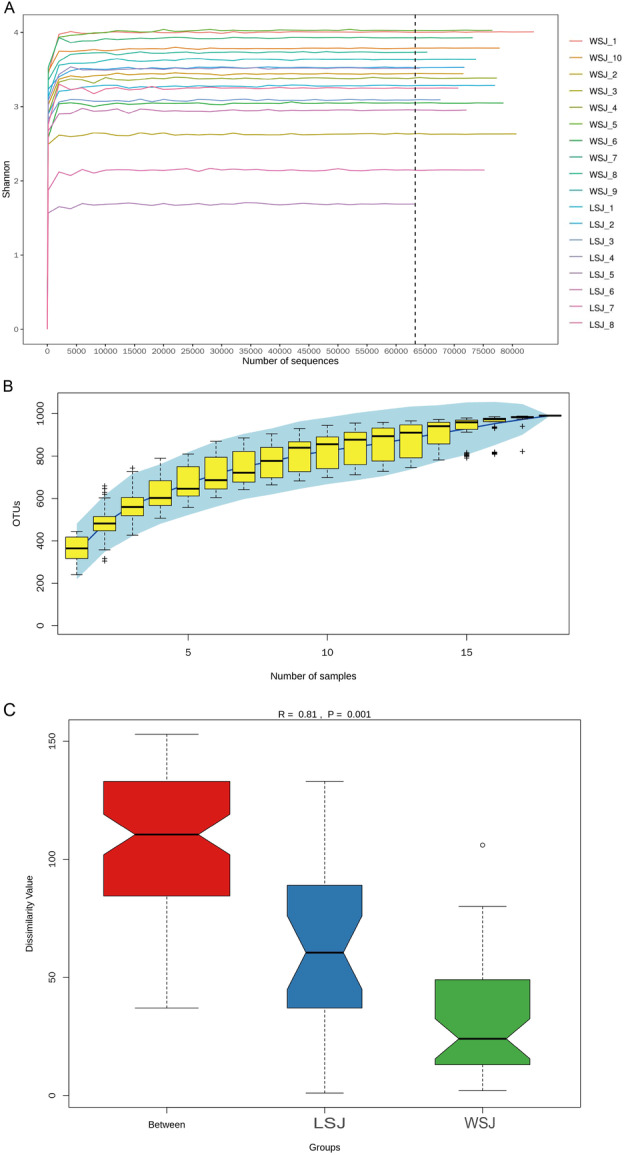
Quality control on the sequencing results. (A) Shannon curve; (B) Species accumulation curve; (C) Anosim analysis (*R* = 0.8095; *p* = 0.001). WSJ Patients with *Schistosoma japonicum*‐induced liver fibrosis living near the infected water, LSJ Patients with *Schistosoma japonicum*‐induced liver fibrosis living on land. ANOSIM analysis of similarities.

Before formally comparing the differences in gut microbiota between the two groups, we conducted quality control on the sequencing results of this study. We first drew the Shannon curve (Figure 1A) and the species accumulation curve (Figure 1B). The Shannon curve was constructed based on the microbial diversity index of the sequencing amount of each sample at different sequencing depths. When the curve tended to be flat, it meant that the amount of sequencing data was large enough to reflect most of the microbial information in the sample. Species accumulation curves were an analysis that describes the increase in species diversity as the sample size increases. It was an effective tool for investigating the species composition of samples and predicting the species abundance in samples. In biodiversity and community surveys, it was widely used to determine whether the sample size was sufficient and to estimate species richness. Our results showed that the amount of sequencing data and sample size in this study could support subsequent analysis and research. In addition, in order to determine whether the grouping of this study was meaningful and whether different grouping factors could explain the differences in samples, we performed Anosim analysis (Figure 1C) and Adonis analysis. Based on the results of Anosim analysis, it was suggested that the differences between groups in this study are significantly greater than the differences within groups, and the grouping was meaningful (*R* = 0.8095; *p* = 0.001). The results of Adonis analysis suggest that different grouping factors in this study could better explain the differences in samples (*R2* = 0.3097; *p* = 0.001).

**Figure 2:**
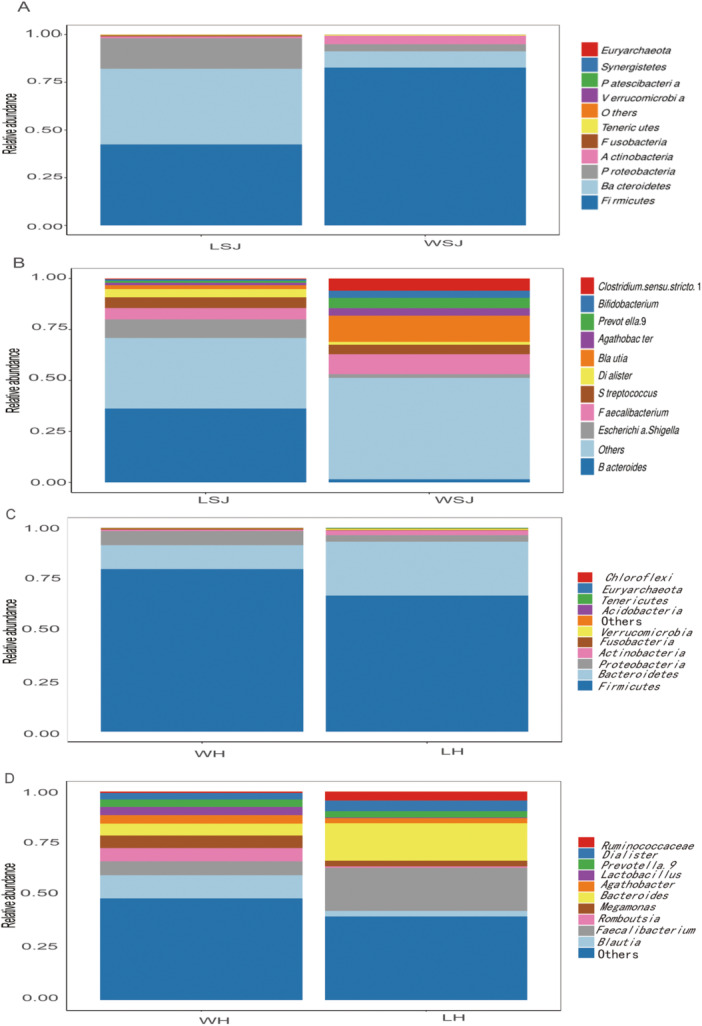
Species community. (A) Phylum level; (B) Genus level. WSJ Patients with *Schistosoma japonicum*‐induced liver fibrosis living near the infected water, LSJ Patients with *Schistosoma japonicum*‐induced liver fibrosis living on land.

**Figure 3:**
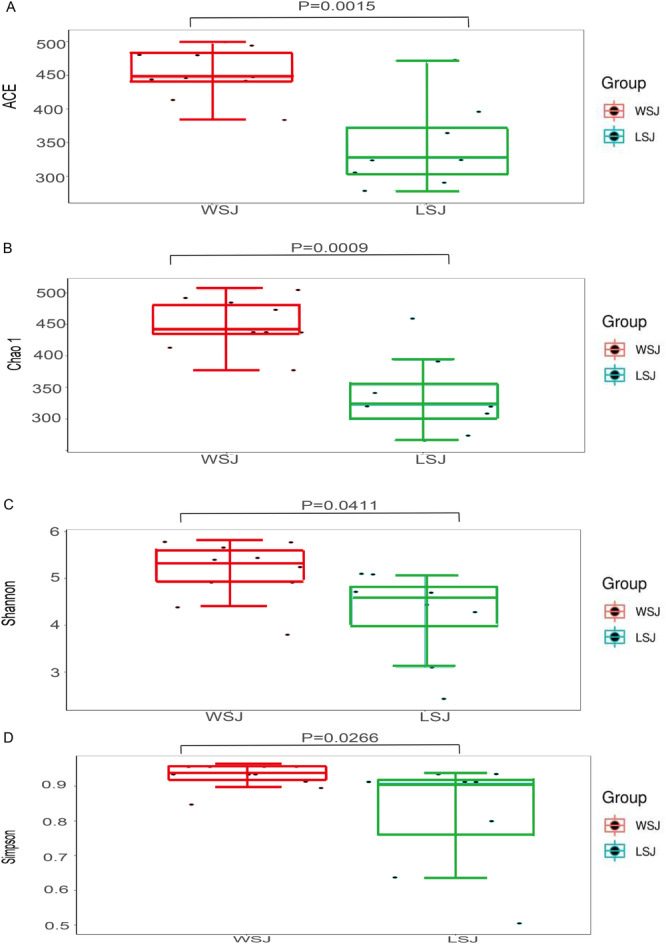
Result of α diversity analysis. (A) Ace index (*t*‐test, *t*_(16)_ = 3.762, *p* = 0.0015), (B) Chao1 index (*t*‐test, *t*_(16)_ = 4.017, *p* = 0.0009), (C) Shannon index (*t*‐test, *t*_(16)_ = 1.816, *p* = 0.0411) (D) Simpson index (*t*‐test, *t*_(16)_ = 2.417, *p* = 0.0266) *WSJ* Patients with *Schistosoma japonicum*‐induced liver fibrosis living near the infected water, *LSJ* Patients with *Schistosoma japonicum*‐induced liver fibrosis living on land.

**Figure 4:**
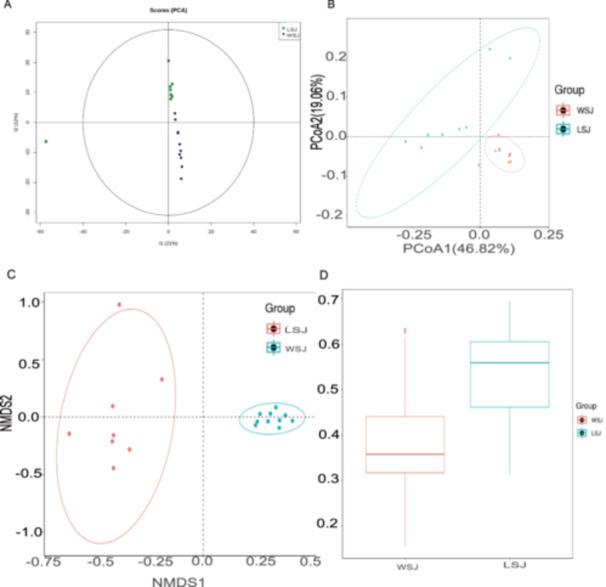
Result of β diversity analysis. (A) PCA; (B) PcoA; (C) NMDS; (D) Beta diversity based on unweighted UniFrac (Wilcoxon, *Z* = −3.302, *p* < 0.001). *WSJ* Patients with *Schistosoma japonicum*‐induced liver fibrosis living near the infected water, *LSJ* Patients with *Schistosoma japonicum*‐induced liver fibrosis living on land. PCA, principal component analysis; PcoA, principal coordinate analysis; NMDS, non‐metric multidimensional scaling.

**Figure 5:**
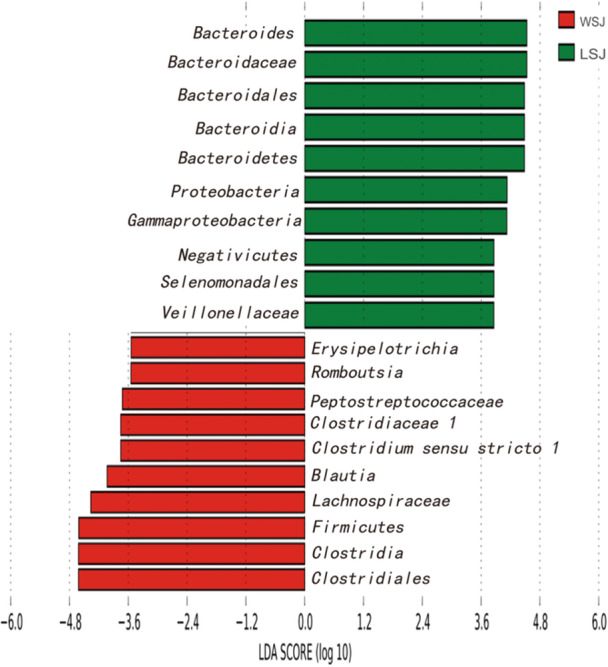
Representative strains. *WSJ* Patients with *Schistosoma japonicum*‐induced liver fibrosis living near the infected water, *LSJ* Patients with *Schistosoma japonicum*‐induced liver fibrosis living on land.

1. **Species community**
  We first conducted a macroscopic study on the species composition of the intestinal flora of the two groups. Based on the species annotation results, we selected the top 10 species with the highest abundance at the Phylum (Figure 2A) and Genus (Figure 3A) levels in each group to draw a species relative abundance column cumulative graph, so as to intuitively view the species with higher relative abundance and their proportions at different classification levels. Our results showed that at the Phylum level, the first and second species of intestinal flora in both groups of patients were *Firmicute* and *Bacteroidetes*. The abundance of *Firmicute* in the intestine of patients living near the infected water was absolutely dominant (82.67%), while the abundance of *Firmicute* (42.41%) and *Bacteroidetes* (39.63%) in the intestine of patients living on land was close. In the WSJ group, *Actinobacteria* (44.41%) was the third most abundant species, while the abundance of *Proteobacteria* (15.9%) in the LSJ group exceeded *Actinobacteria* (9.61%). We further conducted a more detailed investigation at the Genus level, and the species composition of the intestinal flora of the two groups of patients at this level showed great differences. The species with high abundance in the WSJ group were *Blauti* (12.84%), *Faecalibacterium* (9.78%) and *Clostridium. sensu. stricto*. 1 (5.98%). The species with high abundance in the LSJ group were mainly *Bacteroides* (36.2%), *Escherichia shigella* (9.12%) and *Dialister* (4.00%). To determine whether the observed changes in gut microbiota were indeed attributable to the living environment, we further compared the gut microbiota structure of healthy individuals from the two regions. To determine whether the observed changes in gut microbiota were indeed attributable to living environment, we further compared the microbiota structure of healthy individuals from the two regions. At the Phylum level, we found that the top three most abundant species remained consistent in the populations living in the same region. Furthermore, healthy individuals living near the flooded water had higher abundance of Firmicutes and lower abundance of Bacteroidetes compared to those living on land, consistent with observations in the disease group (Figure 2C). At the genus level, although there were significant differences between the healthy and diseased populations, Blautia and Prevotella.9 were both higher in those living near the flooded water than in those living on land (Figure 2D). Therefore, we conclude that differences in living environment are indeed a significant factor contributing to the changes in gut microbiota between the two populations. In summary, although both groups were patients with *Schistosoma japonicum*‐induced liver fibrosis, the species composition of the intestinal flora showed significant differences.
2. **Diversity analysis**
  In the study of intestinal flora, in addition to species composition, alpha diversity (Figure 3) and beta diversity (Figure 4) are also core indicators of ecological analysis, which are used to systematically evaluate the composition characteristics of microbial communities and their changing patterns under different conditions. We continued to explore the diversity of intestinal flora in the two groups. Ace (Figure 3A), Chao1 (Figure 3B), Shannon (Figure 3C) and Simpson indices (Figure 3D) were used to evaluate alpha diversity. The results showed that the diversity of intestinal flora in patients with *Schistosoma japonicum*‐induced liver fibrosis living on land was significantly lower than that in patients with *Schistosoma japonicum*‐induced liver fibrosis living near infected water (Ace index (*t*‐test, *t*_(16)_ = 3.762, *p* = 0.0015), Chao1 index (*t*‐test, *t*_(16)_ = 4.017, *p* = 0.0009), Shannon index (*t*‐test, *t*_(16)_ = 1.816, *p* = 0.0411) Simpson index (*t*‐test, *t*_(16)_ = 2.417, *p* = 0.0266)). PCA (Figure 4A), PcoA (Figure 4B), and NMDS (Figure 4C) analysis were used to compare the beta diversity of the two groups. The results showed that there were also significant differences in beta diversity between the two groups of patients. Unlike the alpha diversity results, the statistical results based on Unweighted Unifra showed that the diversity of intestinal flora in patients with *Schistosoma japonicum*‐induced liver fibrosis living on land was significantly higher than that in patients with *Schistosoma japonicum*‐induced liver fibrosis living near infected water (Figure 4D) (Wilcoxon, *Z* = −3.302, *p* < 0.001). Overall, the species diversity of the two groups also showed significant differences.
3. **Representative strains**
  To further compare the species with important representative significance in the intestinal flora of the two groups, we performed LEfSe analysis. LEfSe is a statistical and machine learning‐based method for identifying microbial markers with significant differences between different groups. It screens differential species through nonparametric tests, quantifies the degree of influence (LDA score) with LDA analysis, and intuitively displays key differential groups through bar charts or evolutionary branch diagrams. We displayed the top 10 differential species in each group (Figure 5). The results showed that the characteristic species of the intestinal flora of patients with *Schistosoma japonicum*‐induced liver fibrosis living on land were *Bacteroide, Proteobacteria*, *Gammaproteobacteria, Negativicutes*, *Selenomonadales*, and *Veillonellaceae*. The characteristic species of the intestinal flora of patients with *Schistosoma japonicum*‐induced liver fibrosis living near infected water were *Clostridiales*, *Clostridia*, *Firmicutes*, *Lachnospiraceae*, and *Blautia*.
4. **Function prediction**

**Figure 6:**
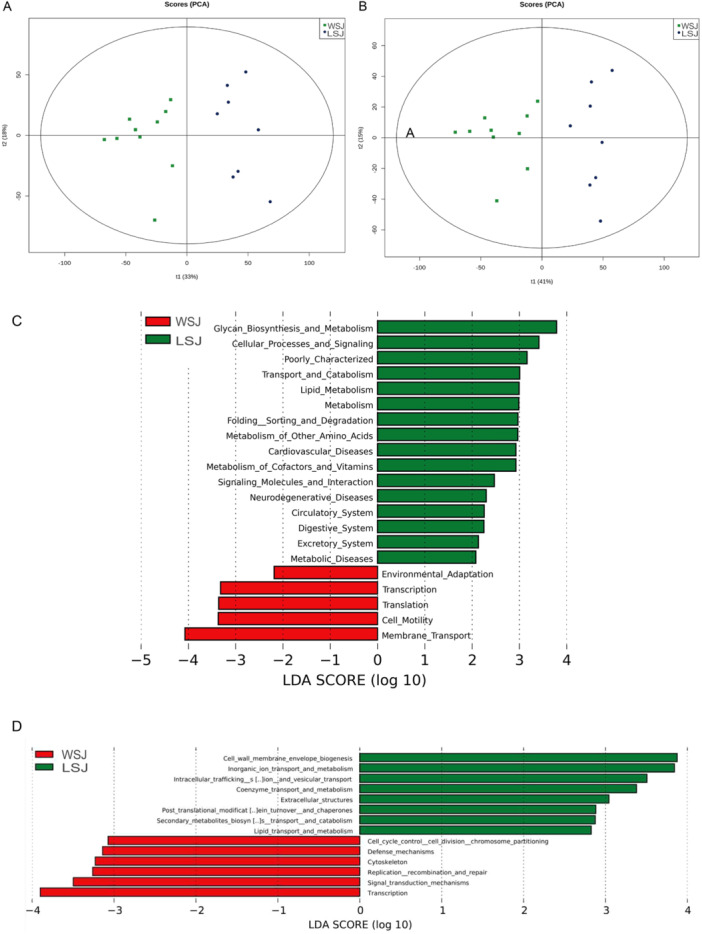
Function prediction. (A) PCA analysis based on the KEGG database; (B) PCA analysis based on the COG database; (C) LEfSe analysis based on KEGG database; (D) LEfSe analysis based on COG database. *WSJ* Patients with *Schistosoma japonicum*‐induced liver fibrosis living near the infected water, *LSJ* Patients with *Schistosoma japonicum*‐induced liver fibrosis living on land. COG, cluster of orthologous groups; KEGG, Kyoto Encyclopedia of Genes and Genomes; LDA, linear discriminant analysis.

Different intestinal flora composition and diversity often indicate that there may be great differences in physiological functions. In order to further clarify the differences in physiological and pathological functions between the two groups, we predicted the functional differences between the two groups based on the results of intestinal flora sequencing combined with the KEGG database and the COG database. PCA analysis showed that there were significant functional differences between the two groups (Figure 6A,B). A more specific analysis based on the KEGG database showed that the physiological function changes of patients with *Schistosoma japonicum*‐induced liver fibrosis living on land were mainly concentrated in Glycan Biosynthesis and Metabolism, Cellular Processes and Signaling, Transport and Catabolism, and Lipid Metabolism. While the physiological function changes of patients with *Schistosoma japonicum*‐induced liver fibrosis living near infected water were mainly concentrated in Environmental Adaptation, Transcription, Translation, Cell Motility, and Membrane Transport (Figure 6C). The results of the COG database analysis showed that the physiological function changes of patients in the LSJ group were mainly in Cell wall membrane envelope biogenesis, Inorganic ion transport and metabolism, and Coenzyme transport and metabolism. The physiological function changes of patients in the WSJ group were mainly in Transcription, Signal transduction mechanisms, and Replication recombination and repair (Figure 6D). The analysis results of the two databases suggested that characteristic changes occurred in the physiological functions of the two groups.

## 4 Discussion

*Schistosomiasis japonica* is an important zoonotic parasitic disease that not only causes irreversible pathological damage to the host, such as liver fibrosis and portal hypertension, but also causes a significant economic burden of the disease (Klotz 2003). China was once one of the countries with the most serious epidemic of the disease, with 11.6 million infected people at the peak (Zhou et al. 2005). Since the 1950s, China has adopted a comprehensive prevention and control strategy based on snail eradication, chemotherapy, fecal management and health education (Cao et al. 2020). Among them, the “fishermen on land” policy (launched in 2004) is a key measure of environmental intervention. It significantly reduces the risk of human exposure by relocating fishermen who have been in contact with infected water for a long time to settle on land. According to statistics from the Hunan Provincial Schistosomiasis Prevention Office, after the implementation of this policy, the infection rate of fishermen along the Dongting Lake decreased from 15.7% in 2003 to 1.2% in 2010. Although this policy has played an important role in curbing the epidemic of schistosomiasis, its long‐term health impact on patients with *Schistosomiasis japonica* cirrhosis is still unknown. Our study explored the impact of this policy on patient health from the perspective of intestinal flora.

Our study first explored the composition of the intestinal flora of patients exposed to infected water and patients living on land. At the phylum level, both groups of patients were dominated by *Firmicutes* and *Bacteroidetes*, but the absolute dominance of Firmicutes in the WSJ group (higher F/B ratio) may reflect the energy metabolism adaptation of those exposed to infected water. Previous studies have shown that an increased F/B ratio is associated with a high‐fat diet or host energy storage needs (Wang et al. 2022). In addition, continuous exposure to infected water may lead to repeated schistosomiasis infection, which may increase intestinal permeability and promote the enrichment of butyrate‐producing *Firmicutes* (such as *Blautia*) to maintain barrier function. In contrast, the balance of *Firmicutes* and *Bacteroidetes* in the LSJ group was closer to that of healthy people, which may be related to the fact that dietary fiber intake (such as vegetables) in their terrestrial living environment promotes the growth of *Bacteroidetes*, which reflects the improvement of the terrestrial living environment on the flora structure (Marques et al. 2017). It is worth noting that the abundance of *Actinobacteria* (including probiotics such as *Bifidobacterium*) in the WSJ group was higher, which may alleviate schistosomiasis‐related inflammation through immune regulation (Sarkar et al. 2024), while the increase of *Proteobacteria* (including potential pathogens such as *Escherichia Shigella*) in the LSJ group indicated intestinal microecological dysbiosis, which may be associated with chronic low‐grade inflammatory state. At the genus level, *Blautia* and *Faecalibacterium* (core bacterial genera producing SCFAs) in the WSJ group may inhibit the NF‐κB pathway through butyrate to reduce intestinal inflammation (Liu et al. 2024). In general, patients who have been exposed to infected water have repeated schistosomiasis infections due to repeated exposure to infected water, and the changes in their intestinal flora structure fully reflect the adaptive changes they have made to resist repeated schistosomiasis infections and intestinal inflammation. This suggests that in future prevention and control work, we need to strengthen the control of repeated schistosomiasis infection in patients who have been in contact with infected water and strengthen the treatment and intervention of intestinal inflammation caused by schistosomiasis infection. From the perspective of risk prediction and early screening, the above differences provide a basis for developing non‐invasive screening tools: the F/B ratio can serve as a rapid screening parameter for high water source exposure risk. Abnormally elevated abundance of Blautia and Faecalibacterium can serve as early warning indicators for high‐risk groups of liver fibrosis. Elevated abundance of Proteobacteria can be used to assess the “latent” inflammatory risk in terrestrial populations. In the future, a multi‐parameter risk scoring system incorporating these indicators can be constructed to achieve stratified patient management—routine follow‐up for low‐risk individuals and priority ultrasound and liver fibrosis screening for high‐risk individuals. Incorporating rapid fecal microbiota detection into the schistosomiasis surveillance system will help shift from “passive treatment” to “active screening.”

In addition to the composition of the microbiota, our study systematically analyzed the intestinal microbiota diversity of the two groups of patients with *Schistosoma japonicum*‐induced liver fibrosis. The lower α diversity of the terrestrial group (LSJ) may reflect the fragility of its intestinal microecological system. This phenomenon is related to the following mechanism: the WSJ group was continuously exposed to a diverse aquatic microbial environment, which may maintain a higher α diversity through “environment‐microbiota” interaction. Supporting evidence can be found in studies that the diversity of intestinal microbiota in fishermen is generally higher than that of land residents. In addition, terrestrial life may be accompanied by more processed food intake (known to reduce diversity), while the high‐fiber diet (such as aquatic plants) of the WSJ group may support the growth of more commensal bacteria. Unweighted Unifrac showed that the LSJ group had a higher β diversity, which may be because the LSJ group had a greater heterogeneity in microbiota composition due to significant individual differences in diet/medication habits. In contrast, the WSJ group had a similar microbiota structure due to common exposure to similar water environments. Our results suggest that we can design personalized probiotic combinations for the LSJ group in the future (to compensate for low α diversity), while for the WSJ group, we should develop ecological regulation therapy based on the high α diversity characteristics of their intestinal flora.

We continued to explore the differences of signature species in the gut microbiota between the two groups, and these findings have important pathophysiological significance. In the terrestrial living group (LSJ group), the enrichment of *Bacteroides* and *Proteobacteria* (especially *Gammaproteobacteria*) may reflect their specific metabolic characteristics and potential inflammatory status (Pittayanon et al. 2019). As the dominant genus in the intestine, *Bacteroides*’ overgrowth may be related to the increased intake of refined carbohydrates in the terrestrial diet (Wu et al. 2011). The enrichment of the Proteobacteria phylum (including a variety of conditional pathogens) is usually regarded as a sign of intestinal dysbiosis, which may aggravate the process of liver fibrosis (Awoniyi et al. 2023). It is worth noting that the significant presence of *Negativicutes*, *Selenomonadales*, and *Veillonellaceae* suggests that patients in the LSJ group may have a special short‐chain fatty acid metabolism pattern, because these species are closely related to lactate metabolism (den Besten et al. 2013). In contrast, the significant enrichment of *Clostridiales*, *Clostridia*, and *Firmicutes* in the infected water contact group (WSJ group) indicates that their intestinal flora has a stronger ability to ferment fiber, which may be due to the richer dietary fiber intake in the infected water environment. In particular, the enrichment of *Lachnospiraceae* and *Blautia* has important functional significance, as these genera are key bacterial communities that produce butyrate (Yang et al. 2024). Butyrate is known to have anti‐inflammatory effects and intestinal barrier protection functions (Guo et al. 2024). This change may be an adaptive change in the WSJ group to cope with the high pressure of repeated *Schistosoma japonicum* infection faced by patients due to continuous exposure to the infected water environment. It is an important manifestation of the body's self‐adjustment to adapt to the environment and resist disease. The identification of these different species provides a new perspective for understanding the impact of different living environments on the intestinal microecology of patients with *Schistosoma japonicum*‐induced liver fibrosis. The enrichment of pro‐inflammatory flora in the LSJ group and the dominance of protective flora in the WSJ group not only reflect the shaping effect of environmental factors on the intestinal flora, but may also partially explain the differences in disease progression in different exposed populations. More importantly, these species may serve as potential biomarkers, providing a theoretical basis for the development of precise microecological intervention strategies for different exposed populations.

Finally, we compared the functional characteristics of the microbiota in patients with schistosomiasis liver fibrosis in the infected water contact group and the terrestrial group. Based on the KEGG database, the terrestrial group (LSJ group) showed significant changes in pathways such as Glycan Biosynthesis and Metabolism and Lipid Metabolism. Combined with the abnormalities in the Cell wall membrane envelope biogenesis and Inorganic ion transport and metabolism pathways shown by the COG database data analysis, it was suggested that this group of patients may have impaired intestinal barrier function and metabolic disorders. In particular, changes in Glycan Biosynthesis and Metabolism may affect the integrity of the mucus layer, increase intestinal permeability, and thus promote endotoxin translocation and liver inflammatory response. In contrast, the enrichment of pathways such as Environmental Adaptation, Transcription, and Membrane Transport in the infected water contact group (WSJ group) reflects the adaptive changes of its microbiota to environmental stress. The active Signal transduction mechanisms and Replication recombination and repair shown in the COG database analysis indicate that the functional changes of the microbiota in the WSJ group focus on enhancing the body's response to environmental stress and genome stability. It is worth noting that the functional differences between the two groups showed a distinct “metabolism‐adaptation” binary classification feature: the LSJ group tended to change the basic metabolic pathway, while the WSJ group focused on environmental adaptation and the improvement of information processing capabilities. This differentiation may be due to different environmental selection pressures: factors such as diet and drugs in terrestrial environments mainly affect the metabolic function of the microbiota, while biotic and abiotic stress factors in the infected water environment drive the adaptive evolution of the microbiota (Flandroy et al. 2018). These findings not only deepen our understanding of the pathogenesis of schistosomiasis microecology, but also provide a theoretical basis for the development of intervention strategies for different exposed populations. From the perspective of precision prevention and control, this binary functional classification of “metabolism‐adaptation” provides a direct basis for stratified intervention strategies. For the LSJ group, characterized by metabolic disorders, “metabolic repair” measures can be prioritized. For example, supplementing with probiotics, prebiotics, and dietary fiber can restore intestinal barrier function and reduce liver inflammation. For the WSJ group, characterized by environmental adaptation, the focus should be on reducing exposure to contaminated water and supplementing with probiotics to maintain the functional stability of the gut microbiota. In addition, future schistosomiasis surveillance systems can incorporate rapid fecal microbiota testing to achieve risk stratification and differentiated follow‐up management for populations with different exposure backgrounds. These findings will drive the transformation of schistosomiasis prevention and control models from a “broad‐spectrum, repetitive chemotherapy” model to a “microbiota typing, precision intervention” model.

Our study compared the differences in intestinal flora between patients with schistosomiasis liver fibrosis who were exposed to infected water and those who lived on land, providing new ideas for understanding the progression of the disease. However, this study has certain limitations. Although our study suggests that the environment and lifestyle and dietary structure may be important factors leading to differences in intestinal flora between the two groups of patients, due to research time and funding limitations, this study was unable to conduct more comprehensive and in‐depth statistics and analysis of the above factors. In addition, there is a lack of further experimental verification of the hypothesis that repeated schistosomiasis infection causes adaptive changes in intestinal flora in patients with infected water contact. Future studies should combine longitudinal cohorts and animal experiments to verify the causality of changes in the flora. Furthermore, future research should integrate metagenomics (to elucidate the functional gene composition of the gut microbiota), metabolomics (to detect key effector molecules such as short‐chain fatty acids, bile acids, and tryptophan metabolites), and host transcriptomics/proteomics (to assess liver and intestinal immune responses). A complete regulatory network of “microbiota‐metabolites‐host targets” should be constructed to elucidate the molecular mechanisms by which the gut microbiota influences the pathological process of schistosomiasis. And develop precise intervention strategies for different exposed populations based on the characteristic flora discovered in this study.

## 5 Conclusion

This study systematically explored the effects of different living environments (contact with infected water vs. living on land) on the intestinal flora of patients with *Schistosoma japonicum*‐induced liver fibrosis under the background of the implementation of China's “Fishermen going ashore” policy. We found that the two groups had significant differences in flora composition, diversity characteristics, iconic species, and functional predictions: the infected water contact group showed characteristics dominated by Firmicutes, high α diversity, enrichment of butyrate‐producing bacteria (such as *Blautia*), and showed enhanced environmental adaptation‐related functions. The terrestrial living group showed an ecological imbalance with increased Proteobacteria and reduced α diversity, accompanied by abnormal glucose and lipid metabolism and impaired barrier function. These differences reflect the adaptive changes of the flora to environmental selection pressure, providing a new perspective to explain the differences in disease progression in different exposed populations. And provide inspiration for the future prevention and control priorities for different populations.

## Author Contributions

**Xiaofei Fan:** conceptualization and methodology. **Chen Zhou:** investigation, writing – original draft preparation, review and editing. **Pengpeng Zhang:** data curation and software. **Yingzi Ming:** project administration and funding acquisition.

## Ethics Statement

The work has been carried out in accordance with The Code of Ethics of the World Medical Association (Declaration of Helsinki) for experiments. All experiments in this study were approved by the Clinical Research Ethics Committee of the Third Xiangya Hospital of Central South University. All participants signed an informed consent form, and patient privacy was fully protected.

## Conflicts of Interest

The authors declared that the research was conducted in the absence of any commercial or financial relationships that could be construed as a potential conflict of interest.

## Data Availability

The data that support the findings of this study are openly available in NCBI Sequence Read Archive at https://www.ncbi.nlm.nih.gov/sra/?term=SRP459542, reference number SRP459542 and PRJNA1018653. The sequencing data have been deposited in the NCBI Sequence Read Archive under the SRP459542 and PRJNA1018653.
